# SMYD Family Members Serve as Potential Prognostic Markers and Correlate with Immune Infiltrates in Gastric Cancer

**DOI:** 10.1155/2023/6032864

**Published:** 2023-02-07

**Authors:** Donghui Liu, Menglin Liu, Wenxin Wang, Xiaoxue Li, Enhong Shi, Chenyao Zhang, Yinghui Wang, Yan Zhang, Liru Wang, Xuyao Wang

**Affiliations:** ^1^School of Life Science and Technology, Harbin Institute of Technology, Harbin 150000, Heilongjiang Province, China; ^2^Department of Oncology, Heilongjiang Provincial Hospital, Harbin 150000, Heilongjiang Province, China; ^3^Department of Pharmacy, Harbin Second Hospital, Harbin 150000, Heilongjiang Province, China

## Abstract

**Background:**

The SMYD family comprises a group of genes encoding lysine methyltransferases, which are closely related to tumorigenesis. However, a systematic understanding of their role in gastric cancer (GC) is lacking.

**Methods:**

Using databases and tools such as the Cancer Genome Atlas, Human Protein Atlas, Kaplan–Meier Plotter, Gene Expression Profiling Interactive Analysis, and Metascape, we comprehensively analyzed differences in SMYD expression and its prognostic value as well as the association of SMYDs with immune cell infiltration, tumor mutational burden (TMB), and microsatellite instability (MSI). We conducted functional enrichment analysis and explored a competing endogenous RNA mechanism regulating SMYD mRNA and protein levels in patients with GC.

**Results:**

In GC, the expression of SMYD2/3/4/5 mRNA was significantly upregulated, as opposed to that of *SMYD1* mRNA, which was significantly downregulated. The protein levels of SMYDs were consistent with mRNA levels. SMYD1/2/4/5 was negatively correlated with overall survival; SMYD1/2/3/5 was negatively correlated with progression-free survival. Our SMYD-based signature and nomogram model may be useful for inferring the prognosis of GC. All SMYDs were closely associated with the infiltration of six immune cell types: uncharacterized, CD8^+^*T*, CD4^+^*T*, macrophage, endothelial, and B cells. TMB was significantly negatively correlated with SMYD1 expression, while a significant positive correlation was observed with SMYD2/5. Furthermore, MSI was significantly positively correlated with SMYD2/5 expression. Long non-coding RNAs, such as chr22-38_28785274-29006793.1, XLOC_002309, and CTD-2008N3.1, were suggested to regulate SMYD expression by sponging multiple microRNAs.

**Conclusion:**

SMYDs are differentially expressed in GC and are thus potential prognostic markers. SMYD expression is closely related to immune infiltration, TMB, and MSI, all of which are closely related to the response to targeted immune therapy.

## 1. Introduction

Gastric cancer (GC) is one of the major causes of cancer-associated death worldwide. According to global cancer statistics released by the World Health Organization, more than 1 million new cases of GC and about 769,000 deaths were reported in 2020. Regarding incidence, GC ranks fifth in occurrence of malignant tumors and fourth based on mortality rate. In East Asian countries, especially China, Japan, and South Korea, the number of new cases is increasing annually [1]. While surgery remains the standard treatment for GC, the disease is often detected in later stages, thereby limiting the efficacy of treatment via surgery alone [2, 3]. Therefore, early diagnosis and accurate prognosis are essential to treatment success. At present, imaging biomarkers are mostly utilized for GC diagnosis and prognosis [4–6], but the complex pathogenesis of GC renders these biomarkers unreliable. Therefore, the identification of reliable predictors and the mechanisms underlying their prognostic value are imperative to diagnosing and prognosticating GC earlier.

The SMYD family comprises genes encoding lysine methyltransferases, which contain a SET domain and MYND zinc finger domain, both of which are closely related to chromatin remodeling, transcription, signal transduction, and cell cycle regulation [7]. In humans, five SMYDs (SMYD1/2/3/4/5) have been reported, and these are involved in several biological processes including the progression of various cancer types [8]. For example, SMYD1 and G6PD can regulate miR-206-mediated rhabdomyosarcoma differentiation through epigenetic and metabolic reprogramming [9]. SMYD2 is closely related to the occurrence and development of multiple cancer types, including cervical, colon, and esophageal cancer [10–12]. SMYD3 is also implicated in cancer progression as it can methylate chromosomal histones, thereby regulating tumor proliferation, apoptosis, invasion, and metastasis [13–15]. Additionally, high SMYD3 expression is associated with poor prognosis [16, 17]. SMYD4 has been identified as a tumor suppressor gene in breast cancer [18], and SMYD5 may be involved in cancer and stem cell maintenance [19, 20]. Finally, several studies have suggested the association of SMYDs with immune infiltration in breast cancer and digestive system malignancies [21, 22].

The occurrence and development of GC are exceptionally complex processes. Studies have suggested that SMYD expression is related to GC prognosis in some patients; however, research on the prognostic value and related mechanisms of SMYDs in GC is lacking [23, 24]. In this study, we conducted a comprehensive analysis of SMYDs based on publicly available databases to determine their potential prognostic value in GC.

## 2. Materials and Methods

### 2.1. SMYD mRNA and Protein Expression Levels in GC and Normal Gastric Tissues

The mRNA expression profile data of 375 GC and 32 normal gastric tissue samples in the Cancer Genome Atlas (TCGA) database (https://portal.gdc.cancer.gov/) were downloaded from the Genomic Data Commons (GDC) website (https://gdc.cancer.gov/). The baseline data sheet is shown in Table 1. Wilcoxon's test was performed in R v4.0.3 to determine the statistical significance of differences between the two groups.

The protein expression levels of five SMYD members in GC tissues and normal gastric tissues were evaluated using immunohistochemical expression data from the Human Protein Atlas (HPA, http://www.proteinatlas.org)—an international project designed to systematically explore the human proteome through antibody-based proteomics [25].

### 2.2. Prognostic Value of SMYDs in GC

Kaplan–Meier Plotter (https://www.kmplot.com) is an online database containing microarray gene expression data and survival information from public databases such as GEO, TCGA, and the European Genome-Phenome Archive; we obtained data of 1440 patients with GC [26]. In the present study, patient samples were divided into two groups (high and low expression) based on the median SMYD expression level. The overall survival (OS) and progression-free survival (PFS) in patients with GC were determined via Kaplan–Meier analysis. A *p* value <0.05 indicated statistical significance.

### 2.3. SMYD Signature-Based Prognostic Model for GC

The RNA-sequencing data of 375 GC tissues obtained from TCGA were combined with the corresponding clinical information of patients. Patient survival was compared via log-rank test and Kaplan–Meier survival analysis. A timeROC analysis was performed to compare the predictive accuracy and risk scores of SMYDs. The least absolute shrinkage and selection operator (LASSO) regression algorithm was used for feature selection, and 10-fold cross-validation was employed to test the accuracy. The above analyses were performed using the “glmnet” package in R.

For the Kaplan–Meier curves, the *p* values and hazard ratios (HRs) with 95% confidence intervals (CIs) were derived via the log-rank test and univariate Cox proportional hazards regression. A *p* value <0.05 indicated statistical significance.

### 2.4. SMYD-Based Nomogram Model for GC Prognosis

We performed univariate and multivariate Cox regression analyses and obtained a forest map using the “forestplot” package in R to display the *p* value, HR, and 95% CI of each variable. Based on the results of the multivariate Cox proportional hazards analysis, we built a nomogram using the “rms” package in R to predict the total recurrence rate in 1 and 3 years. The nomogram provides a graphical representation of these factors, and the prognostic risk of an individual patient can be calculated from the coordinates associated with each risk factor.

### 2.5. Relationship between SMYDs and Immune Cell Infiltration, TMB, and MSI

For a reliable evaluation of immune cell infiltration, we used the R package “immunedeconv,” which integrates six state-of-the-art algorithms: TIMER, xCell, MCP-counter, CIBERSORT, EPIC, and quanTIseq. We then used the EPIC algorithm [27] to analyze the mRNA-sequencing data of 375 GC tissues from TCGA and determine the correlation between SMYDs and the infiltration of seven immune cell types. Thereafter, we used a Spearman correlation analysis to describe the correlation between risk score and the infiltration of six immune cell types as well as the correlation of SMYDs with TMB and MSI (quantitative variables with a non-normal distribution). To this end, we used the R package “ggstatsplot,” and *p*  <  0.05 was considered to indicate statistical significance.

### 2.6. Functional Enrichment and Protein Interaction Network Analysis of SMYDs and Correlated Genes in GC

GEPIA2 (http://gepia2.cancer-pku.cn/#index) is a valuable resource for gene expression analysis of tumor and normal tissue samples from TCGA and GTEx databases. The website provides customizable functions, such as tumor/normal gene differential expression analysis, analysis based on cancer type or pathological stage, patient survival analysis, similar gene detection, correlation analysis, and dimensionality reduction analysis [28]. We used the Correlation Analysis module in GEPIA2 to identify genes with an expression patter similar to that of *SMYDs*. This module searches for genes whose expression patterns are similar to that of another gene or signature in various cancer types.

Metascape (http://metascape.org) is an open, user-friendly, and well-maintained gene list analysis tool that integrates more than 40 types of biological information databases for gene annotation and analysis, ultimately providing a rather unique platform for protein-protein interaction (PPI) network analysis. We used Metascape to annotate and enrich *SMYDs* and 100 genes similar in terms of expression to *SMYDs* [29]. We subjected genes to Gene Ontology (GO) and Kyoto Encyclopedia of Genes and Genomes (KEGG) enrichment analyses using Metascape. Enriched terms with min overlap = 3, *p* value cutoff <0.01, and min enrichment >3 were considered statistically significant. To further define the relationship between terms, a subset of enriched terms was selected and presented as a network graph, where terms with similarity >0.3 were connected by edges. We selected the item with the best *p* value from each of the 20 clusters, limiting each cluster to no more than 15 items and a collective total of no more than 250 items. The network was visualized using Cytoscape [30], where each node represents an enriched term, by first coloring its cluster ID and then its *p* value. The PPI network analysis was performed using the following databases: STRING [31], BioGrid [32], OmniPath [33], and InWeb_IM [33]. Only STRING (physical score >0.132) and physical interactions in BioGrid were used. Furthermore, the Molecular Complex Detection (MCODE) algorithm [34] was applied to identify densely connected network components.

### 2.7. Analysis of Competing Endogenous (ce)RNA Mechanism of SMYD Family Gene Regulation in GC

We used the ENCORI (https://starbase.sysu.edu.cn/) [35] and TargetScan [36] (https://www.targetscan.org/vert_80/) databases to predict micro (mi)RNAs regulated upstream of SMYDs. We input the prediction results in LncBase v3.0 [37] (https://diana.e-ce.uth.gr/lncbasev3/interactions) to further identify the long non-coding (lnc)RNAs regulated upstream of reliable miRNAs and then selected three most reliable lncRNAs per miRNA sample. The regulatory network comprising mRNA, miRNA, and lncRNA was constructed using Cytoscape [30].

### 2.8. Immunohistochemistry (IHC)

Protein expression levels of SMYD2 in GC versus paired paracancerous tissues were assessed using IHC. We used paraffin-embedded samples stored in the pathology department at the Heilongjiang Provincial Hospital affiliated to Harbin Institute of Technology. Eight GC and eight paired paracancerous tissues were acquired, the latter of which were defined as tissues located at least 5 cm away from the edge of the tumor. Sections were incubated with an anti-SMYD2 antibody (AB_10616551, 1 : 200) overnight at 4°C. The percentage of positive cells and staining intensity under the microscope were scored based on semiquantitative results. The number of positive stained cells was determined by observing five high-power fields (×200) and counting the percentage of positive cells. Scores of 0, 1, 2, 3, and 4 points were assigned when the proportions of positive cells were <5%, 5–25%, 26–50%, 51–75%, and 76–100%, respectively. Positive staining intensity was scored as 0 points for colorless, 1 point for pale yellow, 2 points for brownish yellow, and 3 points for dark brownish. The net positive grade was obtained by multiplying the two scores and classified as follows: 0 was not detected, 1–4 indicated low, 5–8 indicated medium, and 9–12 indicated high [38]. All sections were independently scored by two pathologists.

## 3. Results

### 3.1. SMYDs Are Differentially Expressed at the mRNA and Protein Levels in GC and Normal Gastric Tissues

**Figure 1:**
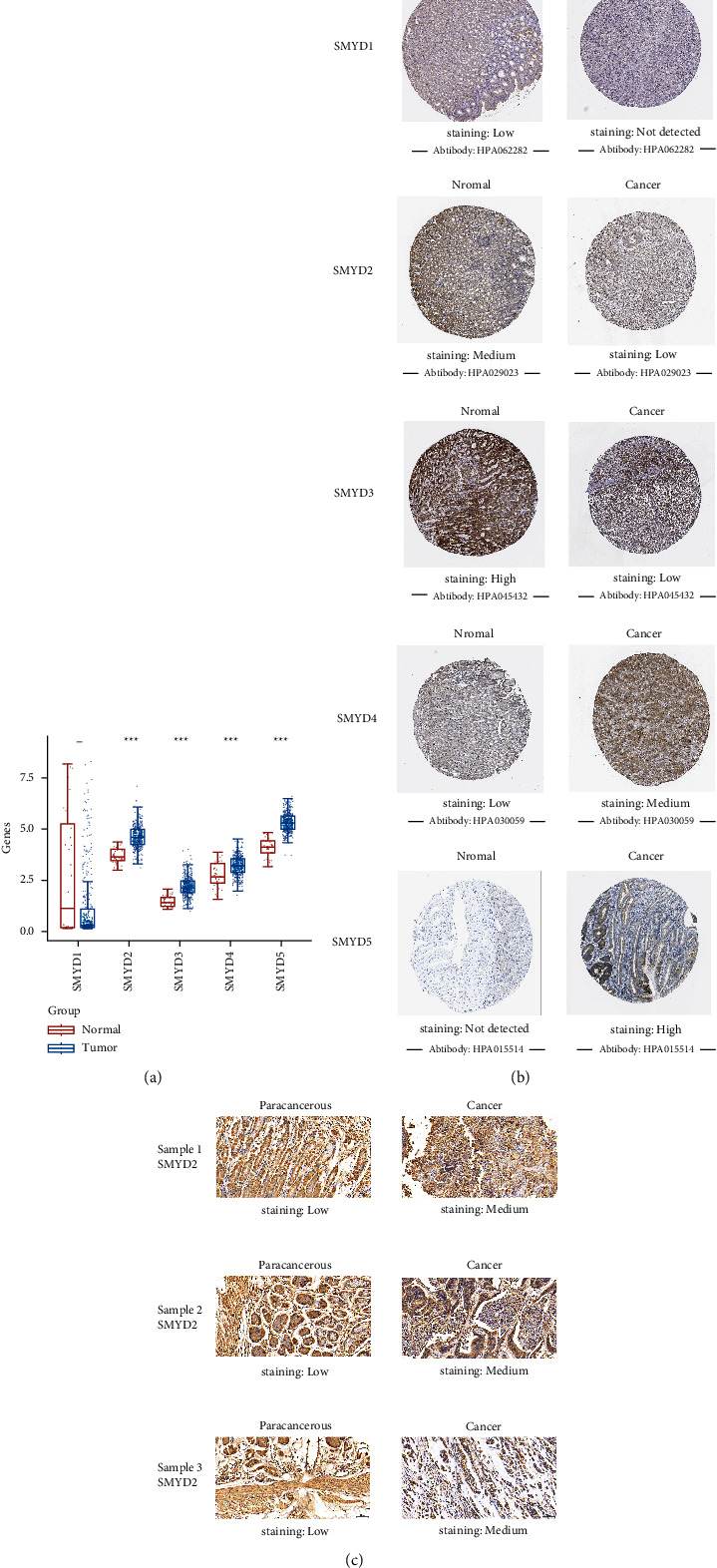
Analysis of mRNA and protein expression levels of SMYDs in gastric cancer and normal gastric tissues. (a) mRNA expression distribution of SMYDs in gastric cancer and normal gastric tissues. The *x*-axis represents the gene name and *y*-axis represents the mRNA expression distribution of the related gene. Red represents the normal gastric tissue group and blue represents the gastric cancer group (^*∗*^*p*  <  0.05, ^*∗∗*^*p*  <  0.01, and ^*∗∗∗*^*p*  <  0.001) (TCGA). (b) Representative immunohistochemical images (HPA) of SMYDs in gastric cancer and normal gastric tissues. (c) Representative immunohistochemical images of SMYD2 in gastric cancer and paired paracancerous tissues (tissue specimen validation). Scale bar, 50 *μ*m; magnification, ×200.

Differential expression analysis was performed on TCGA RNA-sequencing data of 375 patients with GC and 32 normal gastric tissue samples (Figure 1(a)). The expression of *SMYD1* was significantly lower, while that of *SMYD2/3/4/5* was significantly higher in GC tissues compared with that in normal gastric tissues (*p*  <  0.001).

The differential analysis of SMYD protein levels was performed using HPA data (Figure 1(b)). SMYD1 was not expressed in GC but showed low expression in normal gastric tissues. SMYD4 was moderately expressed in GC and showed low expression in normal gastric tissues. SMYD5 was highly expressed in GC but not expressed in normal gastric tissues, consistent with *SMYD1/4/5* mRNA expression. SMYD2 exhibited low expression in GC and moderate expression in normal gastric tissues. SMYD3 also showed low expression in GC but high expression in normal gastric tissues. This result was not consistent with that of *SMYD2* and *SMYD3* mRNA expression. In conclusion, SMYD1/2/3 protein levels were lower and SMYD4/5 levels were relatively higher in GC than in normal tissues.

Immunohistochemical representative images of three pairs revealed moderate SMYD2 expression in GC samples and low expression in paired paracancerous tissues (Figure 1(c)). The other five pairs showed higher SMYD2 expression in GC samples than in the paired paracancerous tissues, except for sample 5 (Supplementary File 1).

### 3.2. Expression of SMYDs Is Significantly Correlated with OS and PFS in Patients with GC

**Figure 2:**
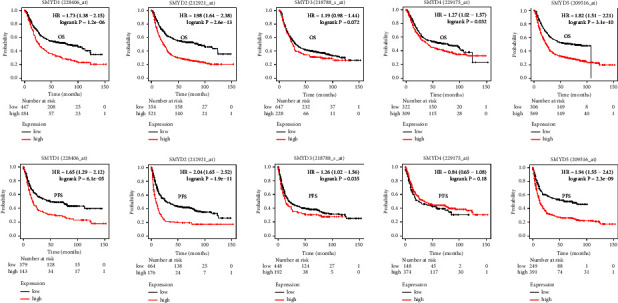
Prognostic value of the mRNA expression levels of *SMYDs* in gastric cancer patients (Kaplan–Meier Plotter). The relationships between high expression (red) and low expression (black) of each *SMYD* mRNA and OS and PFS are presented. Statistical significance is set at *p*  <  0.05.

We conducted a Kaplan–Meier survival analysis based on SMYD levels in patients with GC. SMYD1/2/4/5, but not SMYD3, was significantly associated with OS, which was shorter in the high expression groups (SMYD1 : HR 1.73 [1.38–2.15], *p*=1.2e − 06; SMYD2 : HR 1.98 [1.64–2.38], *p*=2.6e − 13; SMYD3 : HR 1.19 [0.98–1.44], *p*=0.072; SMYD4 : HR 1.27 [1.02–1.57], *p*=0.032; SMYD5 : HR 1.82 [1.51–2.21], *p*=3.1e − 10). Further, SMYD1/2/3/5, but not SMYD4, was significantly associated with PFS, which was shorter in the high expression groups (SMYD1 : HR 1.65 [1.29–2.12], *p*=6.1e − 05; SMYD2 : HR 2.04 [1.65–2.52], *p*=1.9e − 11; SMYD3 : HR 1.26 [1.02–1.56], *p*=0.035; SMYD4 : HR 0.84 [0.65–1.08], *p*=0.18; SMYD5 : HR 1.94 [1.55–2.42], *p*=2.3e − 09) (Figure 2). Taken together, elevated SMYD levels are largely associated with worse GC prognosis.

### 3.3. Signature-Based Prognostic Model Shows That SMYDs Have Potential Prognostic Value in GC

**Figure 3:**
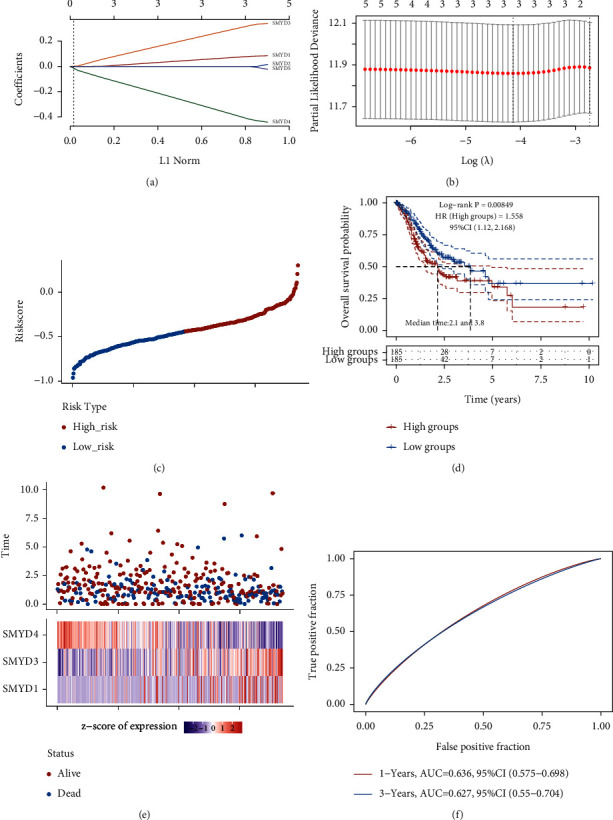
Construction of the SMYD signature prognostic model in gastric cancer. (a) The coefficients of the selected features are represented by the lambda parameter, the *x*-axis represents the value of the independent variable lambda, and the *y*-axis represents the coefficient of the independent variable. (b) The misclassification error of different quantitative variables revealed by LASSO regression model. The red dot represents the misclassification error value, gray line represents the standard error (SE), and the left and right vertical dashed lines represent the optimal value under the minimum criterion and 1-SE criterion, respectively, with “lambda” as the tuning parameter. (c) Risk score, survival time, and survival status in TCGA dataset, the top of which represents the scatter diagram of the risk score from low to high, and different colors represent different expression groups. (d) Scatter plot distribution of survival time and survival status corresponding to the risk score of different samples, in which different groups were subjected to a log-rank test. HR represents the risk coefficient of the samples in the high expression group relative to that of the samples in the low expression group; if HR > 1, the model is a risk factor, but if HR < 1, it means the model is a protective factor. The 95% CI represents the HR confidence interval, and median time represents the median survival time of the high and low expression groups. (e) Expression heatmap of genes in the signature model. (f) ROC curve of 1 and 3-year survival time of the risk model, where the higher the AUC value, the stronger the predictive ability of the model.

The RNA-sequencing and survival data of 375 patients with GC obtained from TCGA were analyzed. The corresponding optimal risk score was determined via LASSO regression (Figures 3(a)–3(c))), using the following formula: risk score = (0.0593) × SMYD1 + (0.2556) × SMYD3 + (−0.3325) × SMYD4. The patients were divided into high and low-risk groups based on median risk score. OS in the high-risk group was significantly shorter than that in the low-risk group (*p*=0.008) (Figure 3(d)). The prognostic signature consisted of SMYD1/3/4 (Figure 3(e)), and the ROC curves of 1 and 3-year survival time had areas under the curve of 0.636 and 0.627, respectively. These results suggest that the model can reliably predict patient survival (Figure 3(f)).

### 3.4. SMYD-Based Nomogram Model Shows That SMYDs Have Potential Prognostic Value in GC

**Figure 4:**
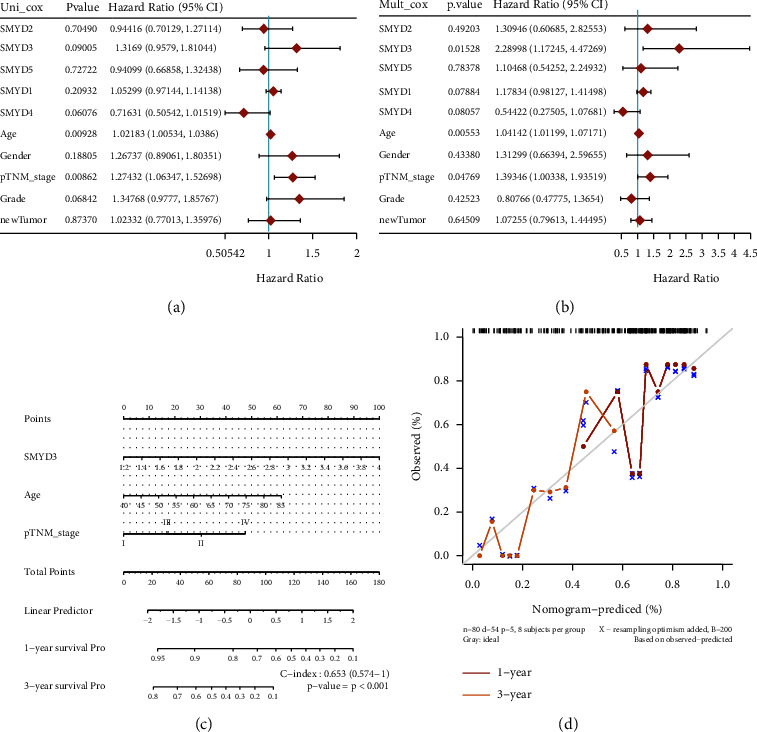
Construction of a nomogram model of SMYDs in gastric cancer. (a, (b) Univariate and multivariate Cox analysis of SMYD mRNA expression levels, *p* values of clinical characteristics, risk factors, and HR with confidence intervals. (c) The nomogram predicts 1 and 3-year overall survival in gastric cancer patients. (d) Calibration curve of the overall survival nomogram model. The diagonal dashed line represents the ideal nomogram, while the red and orange lines represent the observed nomogram for 1 and 3 years, respectively.

*SMYD* mRNA expression profiles were combined with age, gender, pTNM stage, and new tumor event type characteristics in patients with GC. Univariate and multivariate analyses revealed that *SMYD3* mRNA expression, age, and pTNM stage were independent prognostic risk factors (Figures 4(a) and 4(b))). Therefore, we used these factors to construct a nomogram model for predicting 1 and 3-year survival (C-index = 0.653, *p*  <  0.001) (Figure 4(c)). The calibration curve confirmed that our nomogram model has prognostic potential (Figure 4(d)).

### 3.5. SMYD Expression Is Closely Related to Immune Cell Infiltration, TMB, and MSI

**Figure 5:**
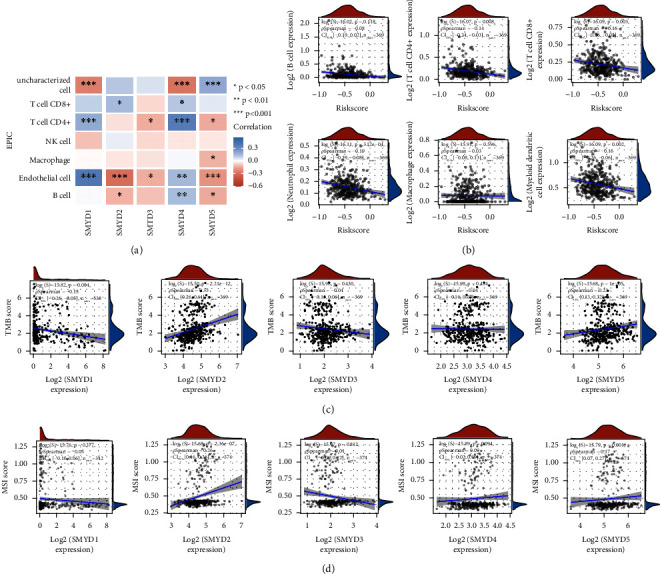
Correlation analysis between SMYDs and infiltration of seven types of immune cells. (a) The *x*-axis in the heat map represents SMYDs while the *y*-axis represents seven types of immune cells, where in red represents a positive correlation and blue represents a negative correlation. Correlation is represented by the color intensity: darker the color, the stronger the correlation between them. The asterisk represents the degree of statistical significance (^*∗*^*p*  <  0.05, ^*∗∗*^*p*  <  0.01, and ^*∗∗∗*^*p*  <  0.001). (b) Correlation analysis between the signature model score and immune cell expression score. In the figure, the *x*-axis represents the risk score distribution, *y*-axis represents the immune score distribution, right density curve represents the immune score distribution trend, upper density curve represents the risk score distribution trend, and the upper value represents the correlation *p* value, correlation coefficient, and correlation calculation method. (c) Correlation analysis of SMYDs and TMB scores. In the figure, the *x*-axis represents the expression level distribution of SMYDs, the *y*-axis represents the TMB score distribution, the right density curve represents the TMB score distribution trend, the upper density curve represents the expression distribution trend of SMYDs, and the upper value represents the correlation *p* value, correlation coefficient, and correlation calculation method. (d) Correlation analysis of SMYDs and MSI scores. In the figure, the *x*-axis represents the expression distribution of SMYDs, *y*-axis represents the MSI score distribution, right density curve represents the MSI score distribution trend, upper density curve represents the SMYD expression distribution trend, and the upper value represents the correlation *p* value, correlation coefficient, and correlation calculation method.

The EPIC algorithm was used to analyze TCGA RNA-sequencing data of GC patients. *SMYD1* expression was positively correlated with uncharacterized cells and negatively correlated with CD4^+^*T* and endothelial cells. *SMYD2* expression was significantly negatively correlated with CD8^+^ T cells but positively correlated with endothelial and B cells. *SMYD3* expression was positively correlated with CD4^+^*T* and endothelial cells. *SMYD4* expression was positively correlated with uncharacterized cells but negatively correlated with CD8^+^*T*, CD4^+^*T*, endothelial, and B cells. *SMYD5* expression was negatively correlated with uncharacterized cells but positively correlated with macrophages and CD4^+^*T*, endothelial, and B cells (Figure 5(a)). The expression of SMYDs was closely associated with the levels of most immune cell types, both positively and negatively, indicating their important role in regulating GC immune microenvironment. In addition, correlation analysis between risk score and immune cell infiltration revealed a negative correlation with CD4^+^*T*, CD8^+^*T*, neutrophil, and myeloid dendritic cells (*p*=0.008, 0.003, 3.12e − 04, and 0.002, respectively) (Figure 5(b)). These results show that our prognostic signature is closely related to immune cell infiltration.

Correlation analysis between SMYDs and TMB scores revealed that TMB was significantly negatively correlated with SMYD1 expression but positively correlated with SMYD2/5 expression (*p*=0.004, 2.21e − 12, and 1e − 05, respectively) (Figure 5(c)). Furthermore, MSI was significantly positively correlated with SMYD2/5 expression (*p*=2.36e − 07 and 0.001, respectively) (Figure 5(d)), indicating that both TMB and MSI are closely related to SMYD expression in GC.

### 3.6. Functional Enrichment and PPI Network Analysis of SMYDs and Similar Genes in GC

**Figure 6:**
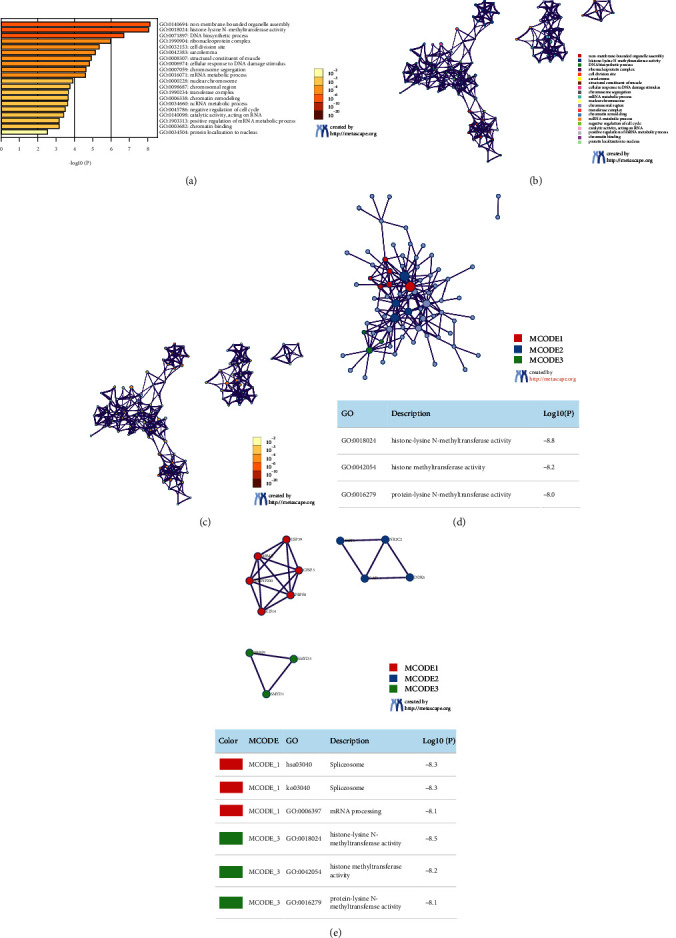
Enrichment analysis of SMYDs and similar gene functions in gastric cancer patients (Metascape). (a) The heat map of GO and KEGG enrichment analysis of SMYDs and 100 genes related to them is colored based on the *p* value. (b, (c) Term-enriched networks: clusters were colored based on cluster IDs, where nodes sharing the same cluster ID are often close to each other, colored based on *p* value, and terms containing more genes tend to have more significant *p* values. (d, (e) Associated protein interaction networks and MCODE components in SMYDs; the pathway and process enrichment analyses were independently applied to each MCODE component, and the three descriptions with the best *p* values were retained as functional descriptions of the corresponding components. The corresponding network diagram is shown in the table below.

Genes correlated to SMYDs based on expression were obtained via the Correlation Analysis module in GEPIA. The top 20 most correlated genes per SMYD family member were selected, resulting in a total of 100 genes (Table 2). We subjected SMYDs and related genes to GO and KEGG enrichment analyses (Figures 6(a)–6(c))) and found that most genes were mainly involved in non-membrane-bounded organelle assembly, DNA biosynthesis, cellular responses to DNA damage stimuli, chromosome segregation, mRNA metabolism, chromatin remodeling, ncRNA metabolism, negative regulation of cell cycle, positive regulation of mRNA metabolism, and protein localization to the nucleus. The enriched molecular functions included participation in histone-lysineN-methyltransferase activity, structural components of muscle, catalytic activity on RNA, and chromatin binding. The enriched cellular component terms included ribonucleoprotein complexes, cell division sites, sarcolemma, nuclear chromosomes, chromosomal regions, and transferase complexes (Table 3). To better understand the mechanisms underlying the relationship between SMYDs and GC, we performed a PPI network analysis. The substantially enriched terms included spliceosome, mRNA treatment, histone-lysine N-methyltransferase activity, histone methyltransferase activity, and protein lysine N-methyltransferase activity (Figures 6(d) and 6(e)).

### 3.7. Analysis of the ceRNA Network of SMYDs in GC

**Figure 7:**
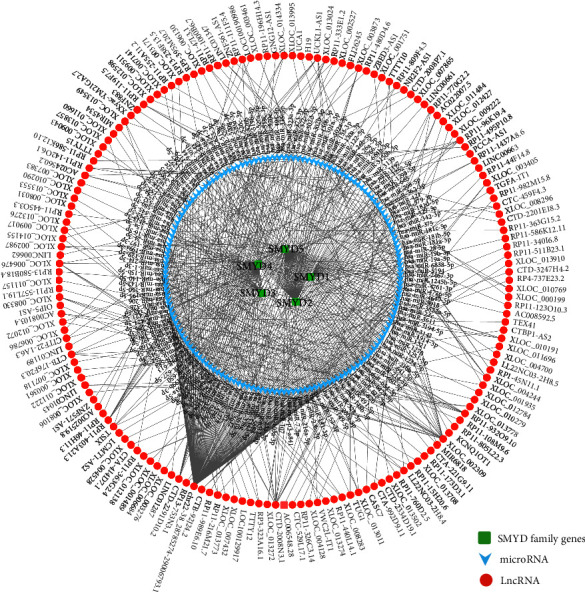
Regulatory network of SMYD ceRNA mechanism in gastric cancer.

A total of 191 reliable miRNAs, which could regulate five mRNAs, were identified. LncBase was used to predict the lncRNAs regulated upstream of reliable miRNAs; we selected the top three most reliable lncRNAs per miRNA. Finally, 466 reliable lncRNAs were obtained, and the mRNA-miRNA-lncRNA regulatory network was constructed (Figure 7).

## 4. Discussion

While SMYDs are known to play important roles in tumor formation, research on their prognostic value and underlying mechanisms in GC has been scarce. Therefore, through public data analysis, we comprehensively explored differences in the expression of SMYD family members and their prognostic merit.

SMYDs have unique tissue specificity and act on both histone and non-histone targets to regulate gene expression and protein activity, with evidence of their involvement in cancer increasing every year [8]. The SMYD1 methyltransferase is specifically expressed in cardiac and skeletal muscle, where it methylates lysine in histone H3, a modification that often occurs at positions K4, K9, K27, K36, and K79. The methylation of histone H4 is mostly observed at K20 [8]. SMYD1 plays a key role in the regulation of embryonic development, cell differentiation, and cardiomyocyte specification [39]. In addition, overexpression of SMYD1 is a high-risk factor in patients with GC [40].

SMYD2 is widely distributed across normal and tumor tissues and, like SMYD1, is involved in cardiac and skeletal muscle cell differentiation and maturation [41]. SMYD2 plays an important role in various cancers; for example, it increases zeste homolog 2 methylation and promotes epithelial-to-mesenchymal transition (EMT) in breast cancer cells [42]. SMYD2 regulates the occurrence and metastasis of RPS7-mediated lung adenocarcinoma, representing itself as a potential prognostic biomarker and therapeutic target [43]. High SMYD2 expression in cervical and liver cancer promotes the proliferation of cancer cells and is considered a risk factor for prognosis [10, 44]. In addition, *SMYD2* knockdown in seven GC cell lines inhibited the proliferation, migration, and invasion of SMYD2-overexpressing cells in a manner independent of *TP53* mutation. Analysis of primary GC tissue specimens revealed that SMYD2 overexpression was positively correlated with tumor size, invasion, lymph node metastasis, and recurrence rate [45]. Overexpressed SMYD2 can methylate *β*-catenin and maintain its stability, thereby activating the Wnt/*β*-catenin signaling pathway to promote GC cell proliferation and metastasis via the EMT [46]. The mechanism of SMYD2-mediated GC metastasis is relatively clear, though identifying the upstream regulatory mechanism is the focus of our next study.

SMYD3 is highly expressed in human platelets and testes [47]. Its roles in cancer include promoting cell proliferation, cell cycle alteration, EMT, increasing telomerase activity, and promoting cell immortalization [48–51]. SMYD3 mRNA and protein expression levels were significantly increased in GC tissues and cell lines relative to that in healthy tissues, with high SMYD3 expression being significantly associated with larger tumor size, lymph node metastasis, and later TNM staging. Further, patients with high SMYD3 expression appear to have a significantly lower 5-year survival rate than those exhibiting low expression [52]. The methylation level of *SMYD3* promoter is significantly lower in colorectal cancer tissues than in adjacent normal tissues. Specifically, *SMYD3* promoter methylation is significantly decreased in patients with lymph node metastasis and stage III/IV disease [53]. Furthermore, SMYD3 is significantly associated with the proliferation, invasion, cell cycle regulation, prognosis, and recurrence of malignant tumors, such as liver, breast, and prostate cancers [54–58].

The results of the present study showed that, in addition to the significantly low expression of *SMYD1* mRNA, SMYD2/3/4/5 mRNAs were significantly overexpressed in GC tissues. The protein expression of SMYD1/2/3 in GC tissues was lower than that in normal gastric tissues, while that of SMYD4/5 was higher. Meanwhile, SMYD2/3 protein expression exhibited a trend contrasting that of mRNA expression, possibly because of the unpaired nature of the GC and normal gastric tissue samples in the HPA database or the existence of post-transcriptional regulatory mechanisms. Therefore, IHC was used to verify the protein expression level of SMYD2 in this study. The results showed that this level was higher in GC tissues than in adjacent normal tissues. Survival analysis revealed that, in general, SMYDs were closely related to OS and PFS in patients with GC, wherein higher expression was associated with worse prognosis. To date, there are relatively few studies on the function of SMYD4 and SMYD5 in cancer, which necessitate further research. The few existing studies have suggested that SMYD4 acts as a tumor suppressor in breast cancer by locally inhibiting platelet growth factor receptor [59], while miR-1307-3p can promote tumor cell proliferation by targeting SMYD4 transcripts [18]. In colon and lung cancer cells, SMYD5 maintains chromosomal integrity by regulating heterochromatin and repressing endogenous repetitive DNA elements during cell differentiation [60]. SMYD5 is differentially expressed in GC, indicating that it is a potential marker for diagnosis and prognosis [19, 20]. By constructing the SMYD-based signature and nomogram model, we confirmed that SMYDs are closely related to GC prognosis and are thus potential prognostic markers.

A close relationship has been described between SMYDs and immune infiltration. SMYD2 is a novel negative regulator of macrophage activation and M1 polarization, whose upregulation inhibits IL-6 and TNF, thereby suppressing the expression of cell surface molecules such as MHC-II and costimulatory factors [61]. Further, H4K20me3 methylation/demethylation catalyzed by SMAD5 and PHF2 regulates the immune balance in vivo, which is essential for inflammatory response regulation [62]. In the present study, we used the EPIC algorithm to analyze TCGA transcriptomic data, which revealed that the expression of SMYDs was closely related to most of immune cells, through which they may regulate the GC immune microenvironment. In addition, a close relationship was observed between the SMYD signature model score and immune cell infiltration, further supporting evidence of a close relationship between SMYDs and immune infiltration. Although TMB and MSI are established as predictive biomarkers of immune checkpoint inhibitor response [63], their association with various potential therapeutic targets and prognostic markers remains unclear. We analyzed the correlation of SMYD expression with TMB and MSI in GC for the first time, confirming that TMB was significantly negatively correlated with SMYD1, while SMYD2/5 was significantly positively correlated with both TMB and MSI. GO and KEGG enrichment analyses and MCODE component analysis revealed that the biological functions of SMYDs and related genes were significantly related to spliceosome, mRNA treatment, histone-lysine N-methyltransferase activity, histone methyltransferase activity, and protein lysine N-methyltransferase activity. After analyzing the ceRNA mechanism of SMYDs, we found that chr22-38_28785274-29006793.1, XLOC_002309, CTD-2008N3.1, and other lncRNAs could modulate SMYD expression by regulating multiple miRNAs. Of these, chr22-38_28785274-29006793.1 might be of the greatest importance, as it is significantly associated with tumor-infiltrating CD4^+^ and CD8^+^ T cells in invasive breast and colon cancers [64, 65]. The regulatory mechanism, however, warrants further validation.

This study explored the differential expression of SMYDs in GC and normal gastric tissues, verifying the protein levels of SMYD2. Using various databases, we comprehensively analyzed the relationships between SMYDs and immune infiltration, TMB, and MSI, in addition to the associated ceRNA mechanisms in GC for the first time. This study was conducted to introduce a more accurate prognostic model for GC. However, our study also has some limitations. For example, we only verified the protein expression of SMYD2 in patient tissues, and the sample size was relatively small. In conclusion, our study shows that SMYDs are differentially expressed in GC, indicating their potential prognostic value, and that they are closely related to immune infiltration, TMB, and MSI.

## Figures and Tables

**Table 1:** Baseline data of patients with gastric cancer (GC).

	Characteristics	GC patients
Status	Alive	228
	Dead	147

Age	Mean (SD)	65.8 (10.7)
	Median [min, max]	67 [35, 90]

Gender	Female	134
	Male	241

Race	Asian	74
	Black	11
	Islander	1
	White	238

pT stage	T1	5
	T1a	2
	T1b	12
	T2	58
	T2a	9
	T2b	13
	T3	168
	T4	30
	T4a	46
	T4b	24
	TX	8

pN stage	N0	111
	N1	97
	N2	75
	N3	26
	N3a	42
	N3b	6
	NX	16

pM stage	M0	330
	M1	25
	MX	20

pTNM stage	I	2
	IA	14
	IB	37
	II	27
	IIA	35
	IIB	49
	III	3
	IIIA	60
	IIIB	52
	IIIC	35
	IV	38

Grade	G1	10
	G2	137
	G3	219
	GX	9

New tumor event type	Metastasis	54
	Primary	3
	Recurrence	29

Radiation therapy	Non-radiation	145
	Radiation	44

History of neoadjuvant treatment	No neoadjuvant treatment	375

Therapy type	Ancillary: chemotherapy	32
	Chemotherapy	128
	Chemotherapy	1
	Chemotherapy: other	1
	Chemotherapy: targeted molecular therapy	1

The measurement data are displayed as mean ± SD. An unpaired *t*-test was used for statistical analysis. The association between GC patients and clinical characteristic variables was analyzed using the Pearson chi-square test or Fisher's exact test.

**Table 2:** Genes with similar expression patterns to *SMYD* family members (GEPIA).

	Similar genes (top 20)
*SMYD1*	*POPDC2, MORN, SYNM, PSD, HAND2-AS1, ACTG2, LINC01573, TPM1, ARHGEF26, CNN1, MYLK, CAP2, LDB3, PGM5, CASQ1, MYH11, PDZRN4, ANGPTL1, CSRP1, DES*
*SMYD2*	*INTS7, C1orf112, CENPF, KIF14, UBE2T, DTL, RRP15, UCHL5, HJURP, NEK2, RCN2, TSEN15, CCT3, EXO1, ILF2, RACGAP1, RBBP5, RBMX, TBCE, USP39*
*SMYD3*	*ACBD6, NVL, H3F3A, SRP9, NUP133, IPO9, INTS7, PIGC, SERPINA11, CENPF, GGPS1, PEG10, RFWD2, RP11-358L22.3, MAP10, PAH, POLR3F, SPRTN, TBCE, TRMT6*
*SMYD4*	*KIAA0753, RPA1, USP22, AKAP10, ANKFY1, THRAP3, SMCR8, RABEP1, ZNF490, AGO3, ZNF445, ZNF740, NR2C2, LATS1, DDX6, NSD1, CELF1, NRF1, PRPF8, ATF7IP*
*SMYD5*	*RTKN, CAD, GTF3C2, PNPT1, CIAO1, PNO1, SMPD4, NOL10, MEN1, ZBTB9, TTI1, PPM1G, CPSF3, GPN1, EIF2B4, E2F6, MEMO1, SNRNP200, ACTR5, HTRA2*

**Table 3:** GO and KEGG functional enrichment analysis results of SMYDs and similar genes in gastric cancer (Metascape).

GO	Category	Description	Count	%	Log_10_ (P)	Log_10_ (q)
GO:0140694	GO biological processes	Non-membrane-bounded organelle assembly	12	11.88	−8.13	−3.99
GO:0018024	GO molecular functions	Histone-lysine N-methyltransferase activity	6	5.94	−8.06	−3.99
GO:0071897	GO biological processes	DNA biosynthetic process	8	7.92	−6.7	−3.33
GO:1990904	GO cellular components	Ribonucleoprotein complex	13	12.87	−5.98	−2.87
GO:0032153	GO cellular components	Cell division site	5	4.95	−5.36	−2.39
GO:0042383	GO cellular components	Sarcolemma	6	5.94	−5.14	−2.21
GO:0008307	GO molecular functions	Structural constituent of muscle	4	3.96	−4.92	−2.06
GO:0006974	GO biological processes	Cellular response to DNA damage stimulus	12	11.88	−4.83	−2
GO:0007059	GO biological processes	Chromosome segregation	8	7.92	−4.64	−1.83
GO:0016071	GO biological processes	mRNA metabolic process	11	10.89	−4.61	−1.81
GO:0000228	GO cellular components	Nuclear chromosome	6	5.94	−3.92	−1.25
GO:0098687	GO cellular components	Chromosomal region	7	6.93	−3.77	−1.15
GO:1990234	GO cellular components	Transferase complex	10	9.9	−3.67	−1.09
GO:0006338	GO biological processes	Chromatin remodeling	6	5.94	−3.66	−1.09
GO:0034660	GO biological processes	ncRNA metabolic process	8	7.92	−3.63	−1.07
GO:0045786	GO biological processes	Negative regulation of cell cycle	7	6.93	−3.5	−0.97
GO:0140098	GO molecular functions	Catalytic activity, acting on RNA	7	6.93	−3.41	−0.92
GO:1903313	GO biological processes	Positive regulation of mRNA metabolic process	4	3.96	−3.17	−0.73
GO:0003682	GO molecular functions	Chromatin binding	8	7.92	−3.17	−0.73
GO:0034504	GO biological processes	Protein localization to nucleus	5	4.95	−2.53	−0.29

The table includes the top 20 clusters and their representative enrichment terms (one per cluster). “Count” refers to the number of genes in the provided list that have membership within the given ontology term. “%” is the percentage of all genes provided found within a given ontology term (only input genes annotated with at least one ontology term are included in the calculation). “Log10(P)” is the *p* value based on Log10. “Log10(*q*)” is a multiple-test adjusted *p* value based on Log10.

## Data Availability

Publicly available datasets were analyzed in this study. These data can be found at TCGA (https://portal.gdc.cancer.gov/), Kaplan–Meier Plotter (https://www.kmplot.com), GEPIA2 (http://gepia2.cancer-pku.cn/#index), Metascape (http://metascape.org), ENCORI (https://starbase.sysu.edu.cn/), TargetScan (https://www.targetscan.org/vert_80/), and LncBase v3.0 (https://diana.e-ce.uth.gr/lncbasev3/interactions). All data, models, and figures generated or used during the study are included within the article.

## Supplementary Materials

Supplementary Materials Supplementary File 1: the immunohistochemical images of SMYD2 in gastric cancer and paired paracancerous tissues (tissue specimen validation); scale bar, 50 *μ*m; magnification, ×200.
