# Supplementary material for: SMYD Family Members Serve as Potential Prognostic Markers and Correlate with Immune Infiltrates in Gastric Cancer
Source: J Oncol. 2023 Feb 7;2023:6032864. doi: 10.1155/2023/6032864 (PMC9929213; doi:10.1155/2023/6032864)
Supplement: Supplementary Materials — Supplementary File 1: the immunohistochemical images of SMYD2 in gastric cancer and paired paracancerous tissues (tissue specimen validation); scale bar, 50 μm; magnification, ×200. [file 6032864.f1.pdf]

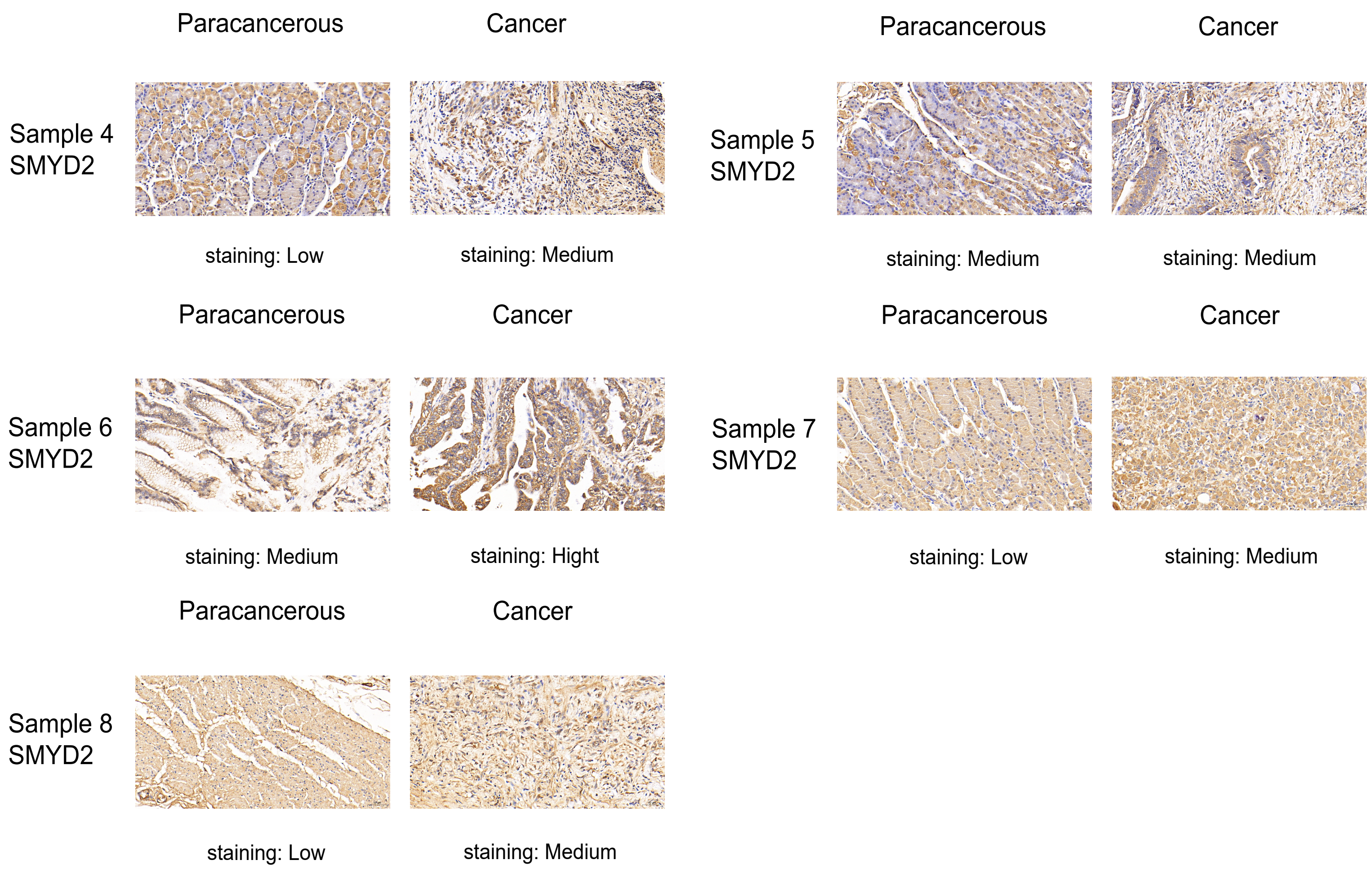
Paracancerous
Cancer
Sample 4
SMYD2
staining: Low
staining: Medium
Paracancerous
Cancer
Sample 5
SMYD2
staining: Medium
staining: Medium
Paracancerous
Cancer
Sample 6
SMYD2
staining: Medium
staining: Hight
Paracancerous
Cancer
Sample 7
SMYD2
staining: Low
staining: Medium
Paracancerous
Cancer
Sample 8
SMYD2
staining: Low
staining: Medium
